# Mechanisms Linking Physical Activity with Psychiatric Symptoms Across the Lifespan: A Systematic Review

**DOI:** 10.1007/s40279-023-01895-0

**Published:** 2023-08-19

**Authors:** Phuong Thuy Nguyen Ho, Pham Bich Tram Ha, Thao Tong, Wichor M. Bramer, Amy Hofman, David Revalds Lubans, Meike W. Vernooij, María Rodriguez-Ayllon

**Affiliations:** 1 Department of Radiology and Nuclear Medicine, Erasmus MC University Medical Center, Rotterdam, The Netherlands; 2 VN-UK Institute for Research and Executive Education, The University of Danang, Da Nang, Vietnam; 3 Erasmus School of Social and Behavioural Sciences, Erasmus University Rotterdam, Rotterdam, The Netherlands; 4 Medical Library, Erasmus MC University Medical Center, Rotterdam, The Netherlands; 5 Department of Epidemiology, Erasmus MC University Medical Center, Rotterdam, The Netherlands; 6 Centre for Active Living and Learning, University of Newcastle, Newcastle, NSW Australia; 7 Faculty of Sport and Health Sciences, University of Jyväskylä, Jyväskylä, Finland

## Abstract

**Background:**

Physical activity has been suggested as a protective factor against psychiatric symptoms. While numerous studies have focused on the magnitude of physical activity’s effect on psychiatric symptoms, few have examined the potential mechanisms.

**Objective:**

The current review aimed to synthesize scientific evidence of the mechanisms through which physical activity might reduce psychiatric symptoms across the lifespan.

**Methods:**

We included articles that were published before March 2022 from five electronic databases (MEDLINE, Web of Science, PsycINFO, Embase, and Cochrane). A qualitative synthesis of studies was conducted. The risk of bias assessment was performed using The Joanna Briggs Institute Critical Appraisal Tool for Systematic Reviews. Studies were included if they explored the possible mechanisms through which physical activity influences psychiatric symptoms (i.e., internalizing and externalizing symptoms) across the lifespan.

**Results:**

A total of 22 articles were included (three randomized controlled trials, four non-randomized controlled trials, three prospective longitudinal studies, and 12 cross-sectional studies). Overall, most of the studies focused on children, adolescents, and young adults. Our findings showed that self-esteem, self-concept, and self-efficacy were the only consistent paths through which physical activity influences psychiatric symptoms (specifically depressive and anxiety symptoms) across the lifespan. There were insufficient studies to determine the role of neurobiological mechanisms.

**Conclusions:**

Overall, future physical activity interventions with the purpose of improving mental health should consider these mechanisms (self-esteem, self-concept, self-efficacy) to develop more effective interventions.

**Clinical Trial Registration:** The protocol of this study was registered in the PROSPERO database (registration number CRD42021239440) and published in April 2022.

**Supplementary Information:**

The online version contains supplementary material available at 10.1007/s40279-023-01895-0.

## Key Points

**Table:** 

Self-esteem, self-concept, and self-efficacy are potential paths through which physical activity might reduce psychiatric symptoms (specifically depressive and anxiety symptoms) across the lifespan. Future studies should consider incorporating strategies to enhance these psychosocial mechanisms in physical activity interventions.
The majority of the studies focused on psychosocial mechanisms while a few studies examined the role of neurobiological mechanisms. It is recommended that future research focus on neurobiological mechanisms.
Integrated studies that examine the combined and independent contributions of the neurobiological and psychosocial mechanisms are needed to obtain the overall picture.
There is a lack of research on externalizing and other internalizing symptoms besides depressive and anxiety symptoms (e.g., somatic symptoms).

## Introduction

According to Lancet Global Health (2020), approximately one billion people around the world are suffering from at least one mental disorder [1]. The economic loss as a result of this was estimated to be $2.5 trillion per year in 2010 and potentially $6 trillion per year in 2030. It is, therefore, not surprising that mental disorders have an enormous impact on many aspects of our lives. While researchers and experts are investigating solutions to these disorders, there is still an urgent need for a better understanding and effective prevention.

Previous studies have shown that most mental health disorders emerged in approximately 50% of individuals by the age of 18 [1]. Psychiatric symptoms can be classified into externalizing and internalizing symptoms. Externalizing symptoms include disinhibited/externally focused behavioral symptoms such as conduct problems, rule-breaking behavior, and attention-deficit/hyperactivity symptoms [2]. Internalizing symptoms include over-inhibited/internally focused symptoms, such as depression, anxiety, and somatic symptoms. Having externalizing and internalizing symptoms in childhood and adolescence can predict mental illnesses later in life [3]. Even though researchers have found many risk factors for internalizing and externalizing symptoms, less has been known about their protective factors [4, 5].

A growing body of research has identified physical activity as a potentially protective factor against psychiatric symptoms and disorders [6]. Physical activity is defined as “any bodily movement produced by skeletal muscle that requires energy expenditure” [7] and has been widely studied because of its extensive health-related benefits [8]. For instance, previous research demonstrated that physical activity has a small-to-moderate effect on mental health; however, the underlying mechanisms responsible for this effect were unclear [9–12]. Understanding the mechanisms linking physical activity with psychiatric symptoms will allow for a better explanation and prediction, and for more effective interventions. This will stimulate the identification of cost-efficient alternative therapies for preventing mental illnesses at all ages.

In 2016, Lubans et al. [13] proposed a conceptual model with three groups of mechanisms (i.e., neurobiological, psychosocial, and behavioral mechanisms) that may explain the effects of physical activity on mental health in children and adolescents. Their study systematically synthesized the existing literature but only found supporting evidence for neurobiological and psychosocial mechanisms. The review only included intervention studies, and although this type of design can provide evidence for cause and effect, observational studies can also provide complementary information, particularly when there is a lack of experimental evidence. In adults, only narrative reviews [13–15] have explored the mechanisms linking physical activity with psychiatric symptoms. In particular, Stillman et al. [8] suggested that physical activity might reduce internalizing symptoms via psychosocial pathways such as mood. Additionally, Kandola et al. [16] proposed biological (e.g., neuroplasticity, inflammation, or oxidative stress) and psychosocial (e.g., self-esteem or social support) mechanisms underlying the relationship between physical activity and depressive symptoms in adults. Previous reviews synthesized the mechanisms through which physical activity can be beneficial in specific clinical populations [17–19]. However, no previous systematic review has synthesized the existing evidence with no age or study-type restrictions in the healthy population.

We aimed to fill the current literature gap by synthesizing the current findings and updating all relevant literature mapping the mechanisms through which physical activity might reduce psychiatric symptoms across the lifespan. In particular, we investigated the two broad categories of mechanisms (neurobiological and psychosocial) that were previously found in the literature [13, 16]. Neurobiological mechanisms were defined as any neurological or biological factor that could explain the effects of physical activity on psychiatric symptoms (e.g., brain functional or structural changes, blood biomarkers). Psychosocial mechanisms were defined as any psychological or social element through which physical activity could have an influence on psychiatric symptoms (e.g., social connection, self-esteem). Additionally, we included both intervention and observational studies and did not impose an age limit to allow for better generalization of our findings. The findings of our review may guide future research to develop more effective treatments and solutions to protect people against mental disorders.

## Methods

Our review adhered to the Preferred Reporting Items for Systematic Review and Meta-Analysis (PRISMA) guidelines [20]. The design of the present work was fully specified in advance. It was registered in the PROSPERO database with the registration number CRD42021239440. Further details on the protocol were made publicly available before conducting the primary electronic search [21].

### Search Strategy and Inclusion Criteria

We searched five electronic databases for relevant articles: MEDLINE All via Ovid, Web of Science Core Collection, PsycINFO via Ovid, Embase via Embase.com, and Cochrane CENTRAL via Wiley (see the Appendix in the Electronic Supplementary Material [ESM] for the full search strategies). Articles included for screening were published before March 2022 (date last searched). In brief, we included articles based on predefined criteria as summarized in the protocol [21]. Two reviewers (PTNH and PBTH) screened the titles and abstracts of the articles separately and included articles based on the inclusion criteria. Any disagreements were discussed and resolved between the two reviewers. Final decisions were made by a third reviewer (MR-A). The eligible articles were retrieved in full text and screened again by the same reviewers to determine full eligibility.

The complete methodology, procedures, and inclusion/exclusion criteria were previously described according to the Participants, Intervention, Comparison, Outcomes, and Setting (PICOS) criteria [23]. Briefly, the inclusion criteria were studies that had: (1) participants, including only humans with at least one group of healthy participants (i.e., no diagnosis of neuropsychological disorders); (2) intervention, physical activity by itself; (3) outcomes, internalizing (i.e., depression, anxiety, somatic symptoms) or externalizing (i.e., conduct problems, rule-breaking behavior, attention deficit/hyperactivity problems) symptoms; and (4) study design, intervention (randomized controlled trial [RCT], non-RCT) and observational studies (prospective longitudinal cohort studies, cross-sectional studies) that explored the neurobiological/psychosocial mechanisms (e.g., mediation) through which physical activity affects psychiatric symptoms. We only included studies that found a beneficial influence of physical activity on psychiatric symptoms because this review focuses on the mechanisms of these beneficial influences. Conference abstracts and other types of gray literature were excluded [22]. Studies that included professional athletes, animals, or only included participants with neuropsychological and/or physical disorders were excluded. We screened further the reference lists of the included studies and contacted an expert in the field (DL) to identify additional studies that may have been missed and any relevant ongoing or unpublished studies.

### Data Extraction

Two researchers (PTNH and TT) extracted the data. This process was double-checked by one experienced researcher (MR-A). Disagreements were discussed between the researchers until a consensus was reached.

We extracted study background (title, author, year, country), sample characteristics (sample size, mean age, percentage of female participants), design (intervention [RCT or non-RCT], or observational [cross-sectional or longitudinal]), independent variables, dependent variables, mediating variables, instruments used to assess the variables, statistical analyses and software, confounders, and main findings. For intervention studies (RCTs and non-RCTs), the time length of intervention, description of the program, intensity, duration, and frequency were also obtained. For longitudinal studies, we also extracted years of follow-up.

### Risk of Bias

The risk of bias of each study was evaluated independently by two researchers (PTNH and TT). Any disagreements were resolved in a consensus meeting with a third researcher (MR-A). The Joanna Briggs Institute Critical Appraisal Tool for Systematic Reviews (https://jbi.global/critical-appraisal-tools) was used to assess the risk of bias. The Joanna Briggs Institute Critical Appraisal Tool for Systematic Reviews includes a checklist with specific criteria to assess the risk of bias for each study design. For each criterion, there are four possible options: “yes” (criterion met), “no” (criterion not met), “unclear,” or “not applicable”. There are eight criteria items for cross-sectional studies, 11 criteria items for longitudinal (or cohort) studies, nine criteria items for non-RCTs (or quasi-experimental studies), and 13 criteria items for RCTs. The appropriateness of the statistical analyses used in these studies was assessed using criteria outlined by Cerin [15]. To classify the risk of bias, we used the method that was previously employed by Molina-Garcia et al. [23]. If at least 75% of the applicable items had been scored as “yes,” then the study was labeled as “low risk”. If less than 75% of the applicable items had been scored as “yes,” then the study was labeled as “high risk”. Any criteria item that was “not applicable” was excluded from the calculation of the percentage.

### Data Synthesis

Findings were synthesized using a method that was previously employed [10, 13, 24]. If 0–33% of studies that reported the same mechanism showed significant results, the mechanism was classified as not significant (Ø); if 34–59% of studies or fewer than four studies reported significant results for the same mechanism, the mechanism was classified as being inconsistent/uncertain (?). Finally, if ≥ 60% of studies found significant results for the same mechanism, the mechanism was classified as significant (✓).

### Modifications to the Initial Protocol

In April 2022, we published the protocol for the current study that outlined our plan to carry out the systematic review in detail [21]. We originally planned to employ the Grading of Recommendations Assessment, Development and Evaluation (GRADE) framework to assess the quality of the evidence across studies. One of the criteria of the GRADE framework was publication bias, which required us to perform a meta-analysis [25]. Because of the heterogeneity of the studies, we were unable to carry out a meta-analysis of the results, and hence, could not employ the GRADE framework. Additionally, because of an increased volume of articles, our initial plan to systematically synthesize the existing evidence of behavioral mechanisms was not implemented.

## Results

### Selection Process

**Fig. 1:**
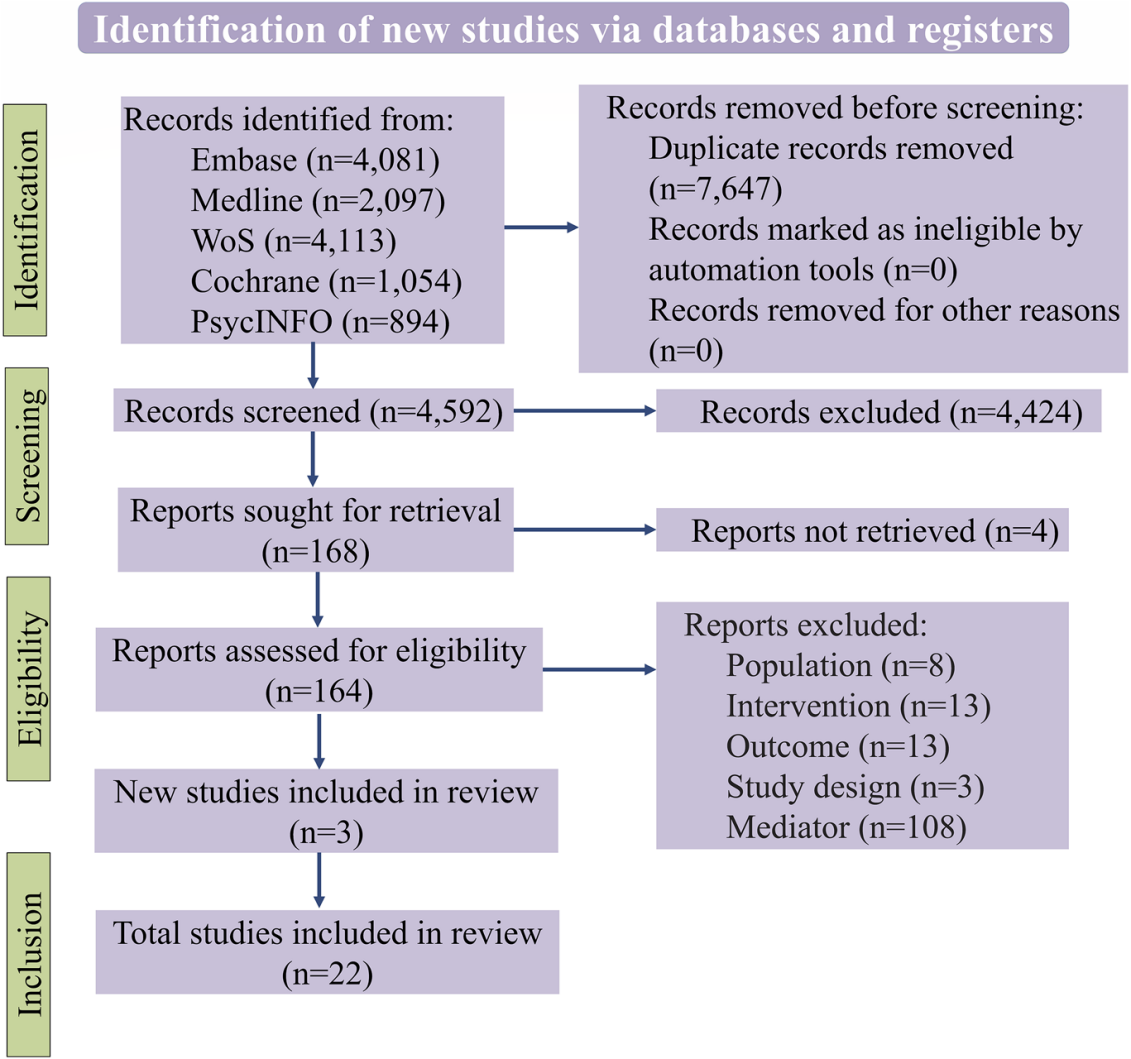
Flow diagram for study selection

The original search of five databases yielded 12,239 articles, 7647 of which were identified as duplicates and removed before screening. There were 4592 remaining articles to screen for titles and abstracts where 4424 articles did not meet the inclusion criteria. This left us with 168 articles for the full-text screening. Out of 168 articles, four articles were unretrievable. There were 145 articles that did not meet the inclusion criteria based on full-text screening (more details in Table S1 of the ESM). Three articles were included after screening the references of the studies that met the inclusion criteria. Finally, 22 articles were included in the review: three RCTs, four non-RCTs, three prospective longitudinal studies, and 12 cross-sectional studies. Further details about the selection process are shown in Fig. 1.

### Summary of Included Studies

A detailed description of the studies included in the systematic review is provided in Table 1.

**Table 1:** Summary of studies investigating the mediator of physical activity and psychiatric symptoms

Study, year (country)	*n* sample (mean age [years] ± SD, % female)	Design; target population	Independent variable (instrument)	Mediating variable (instrument)	Dependent variable (instrument)	Statistical analysis; software	Confounders	Main findings
Alghdir and Gabr, 2020 (Kingdom of Saudi Arabia) [43]	80 (69.6 ± 5.38, 37.5% female)	Non-RCT; elderly individuals	Leisure-time physical activity. Each session is 45–60 min and consists of warm-up, active, and cool-down phases. Three sessions per week for 12 weeks	Adrenal hormones	Depressive mood (Profile of Mood States)	Multiple stepwise regressions, Pearson’s correlation (SPSS)	Data not shown	The findings showed that 12 weeks of supervised exercise interventions are promising non-drug therapeutic strategies in improving depression among older adults. The potential performance in a psychological state occurs physiologically via optimizing the levels of the hormones of the hypothalamus–pituitary–adrenal axis
El Ansari et al., 2011 (UK) [35]	3705 (77.9% female)	Cross-sectional; adolescents, young adults, and adults	Physical activity (“On how many of the past 7 days did you participate in vigorous exercise for at least 20 minutes?”, “On how many of the past 7 days did you participate in moderate exercise for at least 30 minutes?”, “On how many of the past 7 days did you do exercises to strengthen or tone your muscles, such as push-ups, sit-ups, or weight lifting?”)	Body image perception ( studentswere asked: “In your opinion are you …”, with five response options (“Far too thin”, “A little too thin”, “Just right”, “A little overweight”, “Very overweight”), self-rated health status (“How would you rate your health in general?”)	Depression (Modified Beck Depression Inventory)	Regression, stratification analysis (SPSS)	Sex, perceived health, health awareness, muscle strengthening, academic performance	The positive effect of physical activity on depression could be down modulated by the negative impact of a ‘distorted’ body image on depression While low physical activity level was not associated with depression, the protective effect of physical activity increased with physical activity intensity. The association between physical activity and depression may increase with physical activity intensity
Babiss and Gangwisch, 2009 (USA) [27]	14,594 (data not shown, 48.8% female)	Cross-sectional; participants in Wave 1 of the National Longitudinal Study of Adolescent Health (Add Health), adolescents	Sport participation (“During the past week, how many times did you do exercise, such as jogging, walking, karate, jumping rope, gymnastics, or dancing?”)	Social support, self-esteem	Depression (18-item version of the Centers for Epidemiologic Study-Depression Scale) and suicidal ideation (subjects’ yes or no responses to the question: “During the past 12 months, did you ever seriously think about committing suicide?”)	Multivariate hierarchical logistic regression (SAS)	Sex, age, race/ethnicity, receipt of public assistance, and presence of physical limitations that could limit or preclude participation in sports and exercise	The results are consistent with self-esteem and social support acting as partial mediators of the relationships among sports participation and depression As the adolescents’ participation in sports increased, their average self-esteem was progressively higher, and those with higher self-esteem were less likely to suffer from depression
Booij et al., 2015 (NL) [31]	715 (13.5 ± 0.5, 50.9% female)	Longitudinal; adolescents from the Tracking Adolescents’ Individual Lives Survey study with a three-year follow-up	Exercise (participants could specify up to four different types of exercise that they regularly performed, and indicate for every exercise how many days and hours per week they spent on those activities)	Cortisol and heart rate responses to stress (Groningen Social Stress Test), C-reactive protein (immunonephelometric method)	Depressive symptoms (Affective Problems Scale of the Youth Self-Report, a somatic symptoms subscale and an affective symptoms subscale)	Linear regression, indirect method (Preacher and Hayes, 2008)	Sex, social economic status, BMI, total affective or somatic symptom scores, smoking, oral contraceptive use	Exercise was prospectively and inversely related to affective but not somatic symptoms. Heart rate during social stress partially mediated this relationship. No other mediating effects were found
Chae et al., 2017 (South Korea) [37]	848 (data not shown, 58.3% female)	Cross-sectional; adolescents	Physical activity (Physical Activity Questionnaire-Adolescent)	Self-esteem (Body-Esteem Scale For Adolescents and Adults)	Depression (Korean version of Children’s Depression Inventory)	Path analysis, structural equation modeling, AMOS (SPSS)	Sex	Girls showed a significantly higher level of depressive symptoms than boys. Boys showed significantly higher levels of physical activity and body esteem than girls. Body esteem mediated the relation of physical activity with depression
Conley et al., 2020 (USA) [30]	4861 (9.8 ± 0.6, 47.5% female)	Cross-sectional; adults and children	Social physical activity (Adolescent Brain Cognitive Development Sports Activities Involvement Questionnaire)	Social connections (Adolescent Brain Cognitive Development Other Resilience Scale)	Depressive symptoms (The Child Behavior Checklist)	Causal mediation analysis (R mediation package)	Race, age in months, sex, income, site, and family	Number of close friends partially mediated (5.4%, *p* < 0.01) the influence of social physical activity on depressive symptoms. These analyses were replicated in a higher sample of children from the Adolescent Brain Cognitive Development Study (*n* = 7355)
Dishman et al., 2006 (USA) [32]	1250 (17.66 ± 0.61, 100% female)	Cross-sectional; adolescents	Physical activity (3-Day Physical Activity Recall) and sport participation (calculated as the sum of two items adapted from the Youth Risk Behavior Surveillance Survey)	Self-concept (The Physical Self-Description Questionnaire)	Depressive symptoms (The Center for Epidemiological Studies-Depression Scale)	SEM (AMOS)	Physical fitness, BMI	The results provide initial evidence suggesting that physical self-concept mediates the relations of physical activity and sport participation with self-esteem, which is inversely related to depression symptoms among girls in late adolescence
Gorham and Barch, 2020 (USA) [28]	3,973 (120.55 ± 7.32 in months, 48.2% female)	Cross-sectional; children from the Adolescent Brain Cognitive Development Study	Sport involvement (Sports and Activities Involvement Questionnaire)	White matter tract integrity (measured from fractional anisotropy using diffusion magnetic resonance imaging metrics)	Depression (The Child Behavior Checklist)	Generalized linear model	Sex, race, ethnicity, age, parental education, and family income	Involvement in sports was associated with reduced depression in boys. The number of activities and sports that a child was involved in was negatively related to fractional anisotropy of the left fornix but was unrelated to fractional anisotropy of other tracts. Fractional anisotropy of these white matter tracts was also unrelated to depressive symptoms
Gorham et al., 2019 (USA) [29]	4,149 (120.36 ± 7.29 in months, 48.06% female)	Cross-sectional; children from the Adolescent Brain Cognitive Development Study	Sport involvement (Sports and Activities Involvement Questionnaire)	Bilateral hippocampus (magnetic resonance imaging scans)	Depressive symptoms (The Child Behavior Checklist)	Generaliized linear model (SPSS)	Race, ethnicity, age, parental education, family income, and intracranial volume, sex	Sports involvement was positively correlated with hippocampal volume in both boys and girls. Hippocampal volume also interacted with sex to predict depressive symptoms, with a negative relationship in boys, and served as a partial mediator for the relationship between involvement in sports and depressive symptoms in boys
Hamer et al., 2009 (UK) [45]	4,323 (63.4 ± 9.7, 52.6% female)	Longitudinal; elderly individuals from the English Longitudinal Study of Ageing Study with a four-year follow-up	Leisure-time physical activity (interview, the questions about physical activity were extracted from a validated physical activity interview that was employed in the Health Survey for England)	Inflammatory markers (C-reactive protein, fibrinogen)	Depressive symptoms (Centre of Epidemiological Studies Depression Scale)	Logistic regression (SPSS) with and without adjustment for C-reactive protein (potential mediator)	Age, sex, occupational class, baseline depression score, long-standing illness, smoking, alcoholic drinks	Although the inflammatory marker C-reactive protein was predictive of depressive symptoms at the follow-up, it explained less than 5% of the association between physical activity and depression after adjustment for potential confounding factors
Herring et al., 2014 (USA) [39]	1,036 (19.7 ± 2.9 (100% female)	Cross-sectional; young adults	Physical activity (7-Day Physical Activity Recall)	Self-concept (Physical Self-Description Questionnaire), self-esteem (Physical Self-Description Questionnaire)	Anxiety symptoms (Psychiatric Diagnostic Screening Questionnaire)	SEM (Mplus)	BMI, depression symptoms, psychotropic medications	Physical activity had inverse, indirect associations with symptoms of social phobia, generalized anxiety disorder that were expressed through its positive association with specific and global physical self-concept and self-esteem
Hsu et al., 2021 (Taiwan) [36]	64 (16.7 ± 0.8, 62.5% female)	Non-RCT; adolescents	Brisk walking; two instruction/practice sessions at the beginning and at follow-up. Each last 30 min, in one outing or over a number of sessions, but with no one session lasting less than 10 min	Self-concept (Chinese version of the Beck Youth Inventories, Second Edition)	Depression, anxiety (Chinese version of the Beck Youth Inventories-Second Edition)	Regression coefficient, mediation analysis using the causal steps approach suggested by Baron and Kenny (1986) [SPSS version 17]	Data not shown	Adolescents’ self-concept could affect depression by means of altering anxiety as participation in the intervention progressed. Our findings also showed that brisk walking could be effective for diminishing adolescents’ anxiety and depression, especially prior to high school term examinations
Joiner and Tickle, 1998 (USA) [40]	188 (data not shown, 65.9% females)	Cross-sectional; young adults	Exercise ("On average, how many days per week have you exercised during the past 3 weeks?")	Self-esteem (Rosenberg Self-Esteem Questionnaire)	Depression (Beck Depression Inventory)	Regression analyses (to assess mediation criteria of Baron and Kenny, 1986)	Sex	High self-reported exercise level was associated with increases in self-esteem and decreases in depressive symptoms among women. Increases in self-esteem only partly accounted for decreases in depressive symptoms
Kayani et al., 2021 (China) [34]	305 (39.3% female)	Cross-sectional; adolescents, young adults, and adults	Physical actiity (logbooks, self-reports, questionnaires, Cho’s Five-Items Physical Activity Questionnaire, diaries of activity, pedometers, accelerometers, and heart rate monitoring and oxygen consumption)	Self-enhancement (short-form of Self-Enhancement Strategies Scale), self-criticism (Level of Self-Criticism Scale)	Anxiety (short form of the state scale of Charles Spielberger’s State-Trait Anxiety Inventory)	Structural model exhibiting mediation effects by Hayes’ PROCESS version 3 (SPSS)	Age and education	The mediation model supports the mediation of self-enhancement and self-criticism between physical activity and anxiety in university students
Li et al., 2018 (Singapore) [44]	102 (71.4 ± 7.87, 63.7% female)	RCT; elderly individuals	Platform (for exergames), traditional exercise. Each session is one-hour long. One session per week for six weeks	Positive emotions (Positive and Negative Affect Schedule), self-efficacy (The General Self-Efficacy)	Subthreshold depression (9-item Patient Health Questionnaire)	Path analysis (Mplus)	Sex, race, education, living condition	Results confirmed a direct negative platform effect on subthreshold depression and further supported the mediation role of positive emotions in the platform effect. It implied that exergames led to higher positive emotions than traditional exercise, which further reduced the subthreshold depression among older adults. Self-efficacy was not supported to be a significant mediator in the relations between exercise platform and subthreshold depression
Liao et al., 2022 (China) [47]	18 (67.89 ± 4.39; 83.33% female)	Non-RCT; elderly individuals	Tai Chi (One session everyday from 1 to 29 May, 2021. Each session of Tai Chi exercise lasted for one hour, including five min warm-up, 50 min Tai Chi movements and five min cooling down)	BDNF methylation (saliva extracted sample)	Depression (9-item Patient Health Questionnaire)	T-test (SPSS)	Data not shown	Demethylation of BDNF promoter might be one of the potential mechanisms underlying the holistic depression alleviating effect of Tai Chi The potential of BDNF methylation levels as a diagnostic and severity biomarker of depression as well as uncovering the potential epigenetic mechanism of how Tai Chi relieves depressive symptoms
McPhie and Rawana, 2012 (Canada) [33]	4204 (14.7 ± 1.4, 50.2% female)	Longitudinal; adolescents in the National Longitudinal Study of Adolescent Health (Add Health) with a one-year follow-up	Physical activity (three items assessed the frequency of participation in the following activities during the past seven days: (1) rollerblading, roller-skating, skateboarding, and bicycling; (2) playing an active sport, such as baseball, softball, basketball, soccer, swimming, or football; and (3) doing exercise, such as jogging, walking, karate, jumping rope, gymnastics, or dancing)	Self-esteem (Rosenberg Self-Esteem Inventory)	Depressive symptoms (Centers for Epidemiologic Study-Depression Scale)	Mediation analysis (Baron and Kenny, 1986) which is based on regression analyses (SPSS)	Age, race/ethnicity, parent education, and BMI	During early adolescence, self-esteem fully mediated the association between physical activity and depressive symptoms for adolescent boys only. Full mediation was obtained for both boys and girls during late adolescence
Motl et al., 2005 (USA) [46]	174 (65.5, 71.84% female)	RCT; elderly individuals	Walking and toning (resistance and flexibility group). Three sessions per weeks for six months	Physical self-esteem (Physical Self-Perception Profile)	Depressive symptoms (Geriatric Depression Scale)	Latent growth modeling and panel analysis	Data not shown	Depressive symptoms scores were decreased immediately after the intervention, followed by a sustained reduction for 12 and 60 months after intervention initiation; there was no differential pattern of change between the physical activity modes. Change in physical self-esteem predicted change in depressive symptoms
Pickett et al., 2012 (UK) [41]	164 (30, 64% female)	Cross-sectional; young adults to elderly individuals	Total physical activity (International Physical Activity Questionnaire hort Form), leisure time physical activity (Leisure Time Exercise Questionnaire)	Positive and negative affect (Positive and Negative Affect Schedule), coping self-efficacy, physical self-efficacy (Self-Efficacy for Exercise Scale)	Depression (The Beck Depression Inventory-II)	Multiple mediation analyses using the bootstrapping method outlined by Preacher and Hayes (2008)	Age, sex, social support, current treatment and recent negative life events	Improvement in positive affect, pleasant feeling states, negative affect and levels of physical exhaustion significantly mediated the association between leisure-time and total, but not non-leisure time, physical activity and depression. Improvements in physical activity self-efficacy mediated the leisure-time physical activity and depression relationship through improved affect. Coping self-efficacy was not a statistically significant mediator
Ryan, 2008 (USA) [42]	381 (50.66% female)	Retrospective cross-sectional; young adults and adults	Aerobic activity (Physical Activity subscale of the Physical Self-Description Questionnaire)	Self-esteem (Global Self-Esteem subscale of the Physical Self-Description Questionnaire), self-efficacy (Task Efficacy and Scheduling Efficacy subscales of the Physical Self-Description Questionnaire)	Depression (Depression subscale of the Symptom Checklist-90-Revised)	SEM analyses	Data not shown	These findings are consistent with the proposed model and imply that independent self-esteem and self-efficacy mechanisms are sufficient to account for the antidepressant effects of physical activity
White et al., 2009 (UK) [26]	39 (21, 82.1% female)	Non-RCT; young adults	Physical activity (structured weekly diaries). Two to three sessions per week. Each session lasts 30 min. The program lasts eight weeks	Positive affect and negative affect (Positive and Negative Affect Schedule), Self-esteem (Rosenberg’s Global Self-Esteem Scale), physical self-perceptions (Physical Self-Perception Profile), physical self-efficacy (Perceived Physical Ability subscale of the Physical Self-Efficacy Scale)	Depression (The Beck Depression Inventory-II)	ANOVA, intention-to-treat (last observation carried forward approach)	Data not shown	Change in positive affect, negative affect and self-efficacy present stronger candidate mechanisms than change in self-esteem and self-perceptions for mediating change in depression, at least in the early stages of increased activity. An increase in positive affect may be especially important
Wipfli et al., 2011 (USA) [38]	65 (20.66 ± 2.1, 76.9% female)	RCT; young adults	Stationary cycling and stretching. Each session is 30 min long. Three sessions per week for seven weeks	Self-concept and self-esteem (Physical Self-Description Questionnaire), serotonin level (ELISA), self-efficacy (the Exercise Self- Efficacy Scale)	Anxiety (The State-Trait Anxiety Inventory), Depression (Beck Depression Inventory)	Linear regression analyses (to test mediation criteria by Judd and Kenny, 1981)	Data not shown	Change in serotonin was determined to partially mediate the relationship between exercise and depression. Self-concept, self-esteem, and self-efficacy were not found to mediate the relationship between exercise and depression

*AMOS* analysis of moment structure, *ANOVA* analysis of variance, *BDNF* brain-derived neurotrophic factor, *BMI* body mass index, *ELISA* enzyme-linked immunosorbent assay, *NL* The Netherlands, *Non-RCT* non-randomized controlled trial, *RCT* randomized controlled trial, *RUSATED* Sleep Regularity, Satisfaction with sleep, Alertness during waking hours, Timing of sleep, Sleep Efficiency, Sleep Duration, *SAS* Statistical Analysis System, *SD* standard deviation, *SEM* structural equation modeling, *SPSS* Statistical Package for Social Sciences statistical software

#### Characteristics of Included Studies

The sample size of the studies ranged from 18 [47] to 14,594 [27]. Three studies included children [28–30], eight studies included adolescents [26, 27, 31–37], eight studies included young adults [26, 34, 35, 38–42], four studies included adults [34, 35, 41, 42], and six studies included elderly people [41, 43–47].

#### Exposure Characteristics

Out of 22 studies, four studies used sports participation [27–29, 32], five studies used exercise [31, 38, 40, 44, 47], and 14 studies used physical activity as the predictor [26, 30, 32–37, 39, 41–43, 45, 46]. Three intervention studies included an active control group [38, 44, 46]. Within these studies, the experimental conditions included exergames [44], resistance and flexibility exercise [46], and cycling [38]. Three intervention studies did not have a control group; all participants had to participate in the same training program [36, 43, 47]. Session duration ranged from 30 min [38] to 60 min [43, 44, 47]. The frequency of the session ranged from one [44] to seven [47] times per week. Lastly, the overall duration of the interventions varied between four [47] and 24 weeks [46].

#### Mechanism Characteristics

Within the 17 studies that explored the psychosocial mechanisms linking physical activity with psychiatric symptoms, two studies used social support/connection [27, 30], one study used body image [35], one study used self-criticism and self-enhancement [34], eight studies used self-esteem [26, 27, 33, 37–39, 42, 46], four studies used self-concept [32, 36, 38, 39], five studies used self-efficacy [26, 38, 41, 42, 44], one study used self-perception [26], and three studies used mood [26, 41, 44] as the potential mechanisms of interest. Seven studies explored neurobiological mechanisms including hippocampal volume [30], white matter microstructure [28], stress-induced biomarkers [31], neurotransmitters [38], hormones [43], inflammatory biomarkers [31, 45], and brain-derived neurotrophic factor (BDNF) methylation [47].

#### Outcome Characteristics

Depression was used as an outcome variable in 20 studies [26–33, 35–38, 40–47]. One study used the Profile of Mood States [43], three studies used the Center for Epidemiological Studies-Depression Scale [32, 33, 45], one study used the 18-item version of the Center for Epidemiologic Study-Depression Scale [27], one study used the Affective Problems Scale of the Youth Self-Report [31], one study used the Korean version of Children’s Depression Inventory [37], three studies used the Child Behavior Checklist [28–30], two studies used the Beck Depression Inventory [38, 40], two studies used the 9-item Patient Health Questionnaire-9 [44, 47], one study used the Geriatric Depression Scale [46], two studies used the Beck Depression Inventory-II [26, 41], one used the Chinese version of the Beck Youth Inventories-Second Edition [36], one study used the Modified Beck Depression Inventory [35], and one study used the Depression Subscale of the Symptom Checklist-90-Revised [42]. One study also assessed the affective and somatic symptoms of depression [31].

Four studies used anxiety symptoms as the outcome variable [34, 36, 38, 39]. In particular, one study used the State-Trait Anxiety Inventory [38], one study used the short form of the State Scale from the Charles Spielberger’s State-Trait Anxiety Inventory [34], one used the Chinese version of the Beck Youth Inventories, Second Edition [36], and one used the Psychiatric Diagnostic Screening Questionnaire [39].

### Synthesis of Findings

The qualitative synthesis of our findings is summarized in Tables 2 and 3. Overall, there was consistent evidence for three mechanisms linking physical activity with psychiatric symptoms: self-esteem (eight out of ten studies, 80%), self-concept (three out of four studies, 75%), and self-efficacy (three out of five studies, 60%). There were insufficient studies (fewer than four studies) to determine the role of social support, body image, self-criticism, self-enhancement, self-perception, mood, hippocampal volume, white matter microstructure, stress-induced markers, neurotransmitters, hormones, inflammatory markers, and BDNF methylation in the relationship between physical activity and psychiatric symptoms across the lifespan.

**Table 2:** Qualitative synthesis of neurobiological mechanisms linking physical activity with psychiatric symptoms

Study, year	*N*	Age (years)	Hippocampal volume	White matter microstructure	Stress biomarkers	Inflamatory biomarkers	Hormones/neurotransmitters	BDNF methylation
**Cross-sectional**								
Gorham and Barch, 2020 [28]	3973	9–11		Ø				
Ryan, 2008 [42]	381	NT						
Gorham et al., 2019 [29]	4149	9–11	✓♂ Ø ♀					
**Longitudinal**								
Booij et al. 2015 [31]	715	NT			✓	Ø		
Hamer et al., 2009 [45]	4323	NT				✓		
**RCT**								
Wipfli et al., 2011 [38]	65	NT					✓	
**Non-RCT**								
Liao et al., 2022 [47]	18	NT						✓
Alghdir and Gabr, 2020 [43]	80	65–95					✓	
**Summary**								
✓			0.5		1	1	2	1
Ø			0.5	1	0	1	0	0
Total score			?	?	?	?	?	?

*BDNF* brain-derived neurotrophic factor, *Non-RCT* non-randomized controlled trial, *NT* data not shown, *RCT* randomized controlled trial, ? inconsistent/uncertain, ✓ significant, Ø no association, ♂ male, ♀ female

**Table 3:** Qualitative synthesis of psychosocial mechanisms linking physical activity with psychiatric symptoms

Study, year	*N*	Age (years)	Social connection	Body image	Self-criticism	Self-enhancement	Self-esteem	Self-concept	Self-efficacy	Self-perception	Mood
**Cross-sectional**											
Babiss and Gangwisch, 2009 [27]	14,594	11–21	✓				✓				
Chae et al., 2017 [37]	848	15–18					✓				
Conley et al., 2020 [30]	341	NT									
	4520	9–11	✓								
Dishman et al., 2006 [32]	1250	NT					✓	✓			
El Ansari et al., 2011 [35]	2705	NT		✓							
Kayani et al., 2021 [34]	305	18–36			✓	✓					
Ryan, 2008 [42]	381	NT					✓		✓		
Herring et al., 2014 [39]	1036	16–46					✓	✓			
Joiner and Tickle, 1998 [40]	188	NT					✓				
Pickett et al., 2012 [41]	164	19–63							✓		✓
**Longitudinal**											
McPhie and Rawana, 2012 [33]	4204	NT					✓				
**RCT**											
Li et al., 2018 [44]	102	NT							Ø		✓
Motl et al., 2005 [46]	174	NT					✓				
Wipfli et al., 2011 [38]	65	NT					Ø	Ø	Ø		
**Non-RCT**											
Hsu et al., 2021 [36]	64	NT						✓			
White et al., 2009 [26]	39	18–45					Ø		✓	Ø	✓
**Summary**											
✓			2	1	1	1	8	3	3	0	3
Ø			0	0	0	0	2	1	2	1	0
Total score			?	?	?	?	✓	✓	✓	?	?

*Non-RCT* non-randomized controlled trial, *NT* data not shown, *RCT* randomized controlled trial, ? inconsistent/uncertain, ✓ significant, Ø no association, ♂ male, ♀ female

### Risk of Bias Assessment

The detailed risk of bias assessment is included in Tables S2–S9 of the ESM. The criteria to assess the risk of bias and the percentage of studies that met the criteria item by item are presented in Tables S2–S5 of the ESM. The risk of bias assessment study by study is presented in Tables S6–S9 of the ESM. First, all three RCTs showed a high risk of bias [38, 44, 46]. Second, half of the non-RCTs also showed a high risk of bias [26, 47]. Third, one out of three longitudinal studies showed a low risk of bias [33, 45]. Last, of the 12 cross-sectional studies, ten showed a high risk of bias [28–30, 32, 35, 37, 39–42], and two studies showed a low risk of bias [27, 34].

## Discussion

The current systematic review aimed to synthesize the existing literature on the mechanisms through which physical activity reduces psychiatric symptoms across the lifespan. In brief, most of the studies focused on psychosocial mechanisms. They consistently showed that self-esteem, self-concept, and self-efficacy are pathways through which physical activity reduces internalizing symptoms (i.e., depressive symptoms) in the healthy general population, mainly in young people. We found that only a limited number of studies explored the role of neurobiological (e.g., gray matter volume in the hippocampus) mechanisms mainly in youth, making it difficult to obtain the overall picture. Therefore, future studies are encouraged to focus on: (1) exploring the neurobiological mechanism linking physical activity with psychiatric symptoms and (2) building a comprehensive and integrative model that includes all potential mechanisms. For instance, Rodriguez-Ayllon et al. [48] investigated an integrated model including neurobiological, psychosocial, and behavioral mechanisms in children. They were able to establish self-esteem as a mediator for the relationship between sports participation and internalizing symptoms.

### Psychosocial Mechanisms

While a variety of psychosocial mechanisms were identified in this systematic review, only self-esteem, self-concept, and self-efficacy were found to be mediators of physical activity and psychiatric symptoms. This finding aligns with previously proposed mechanisms and models such as Sonstroem and Morgan’s exercise and self-esteem model or Shavelson et al.’s self-concept model [13, 16, 49, 50]. It is noteworthy that self-esteem, self-concept, and self-efficacy are all related to the structure of the self with self-concept being the broad awareness of ourselves while self-concept and self-efficacy are the evaluations/beliefs we have about ourselves and our ability. These self-structures also associate with a wide range of psychiatric symptoms such as depressive and anxiety symptoms. Doing physical activity often has a beneficial impact on these self-structures, which helps alleviate psychiatric symptoms.

The impact of self-esteem and self-concept mechanisms can be attributed to physical self-esteem and physical self-concept. Doing physical activity will not only have a significant impact on our physical health and appearance but also improve the personal concept and evaluations of our physique, which will improve our self-concept and self-esteem as well as psychiatric symptoms, especially in female individuals [27, 37, 39]. Chae et al. [37] recommended several interventions that healthcare professionals could employ to target these mechanisms such as education on body esteem at an early age and raising public awareness through improving mass media literacy. Vella et al. [51] also suggested delivering organized physical activity sessions that target basic psychological needs through appropriate instructional styles. Specifically, they recommended taking into account participants’ preferences, providing a variety of options, and reducing potential pressure.

Self-efficacy mechanisms include ‘task efficacy’ and ‘scheduling efficacy’ [52, 53]. ‘Task efficacy’ is the perception that an individual has of his/her ability to perform a specific task in a specific situation. It assumes that different physical tasks/activities may provide different beneficial effects and these benefits cannot be generalized to other areas. ‘Scheduling efficacy’ suggests that the beneficial effect of physical activity stems from goal setting, adherence to routine, and coping with difficulties, and can generalize to other domains. Ryan found some evidence that supports the mediation effect of self-efficacy, specifically ‘scheduling efficacy’ [42]. Therefore, this author suggested utilizing the self-efficacy mechanism through tangible actions such as helping participants set realistic goals, identifying appropriate physical activity, and learning how to follow exercise goals and progress.

Most of the studies that investigated psychosocial mechanisms were observational and very few were interventions. Only three out of ten studies that investigated self-esteem were interventional studies and only one study found a mediation effect of self-esteem, but this study had a high risk of bias [46]. Only one interventional study found evidence for self-efficacy, but this study did not utilize the appropriate statistical analysis [26]. Therefore, more evidence from intervention studies is needed to better support the mediation effects of self-esteem, self-concept, and self-efficacy.

In addition to these mechanisms, there was evidence that other psychosocial factors (i.e., social connections, body image, self-criticism, self-enhancement, self-perception, and mood) may also mediate the relationship between physical activity and psychiatric symptoms [26, 27, 30, 32, 34, 35, 38, 41, 44]. Most of these psychosocial factors belong to self-structures such as global self-concept and self-esteem, and hence, could potentially have similar mechanisms [16]. However, there were not enough studies to draw strong conclusions.

### Neurobiological Mechanisms

The role of the neurobiological mechanisms in the relationship between physical activity and psychiatric symptoms was unclear because of the inconsistencies and heterogeneity found in this review. These mechanisms can be split into two main categories: brain biomarkers and blood biomarkers.

The brain biomarker studies included in this review only considered the role of brain structures including hippocampal volume, white matter microstructure in the fornix, and the parahippocampal cingulum [28, 29]. It is noteworthy that these studies investigated depressive symptoms in pre-adolescents using the data from the Adolescent Brain and Cognitive Development (ABCD) study [28, 29], which could have resulted in more robust results than expected because of the large sample size. Previous studies have established the link between physical activity, depressive symptoms, and brain abnormalities, particularly of the hippocampus [16, 19]. Studies that focused on depressed individuals also linked changes in functional brain activity to depression as a result of physical activity; however, this phenomenon was only found in the older population [19, 54]. In this review, we did not find any studies that investigated functional brain changes, and this could be a potential topic for future research.

In healthy young individuals, neurobiological measurements in the form of blood biomarkers could provide a more dynamic indication of the role of neurobiological mechanisms in the relationship between physical activity and psychiatric symptoms across the lifespan. For example, stress and inflammatory blood biomarkers, which have been previously linked to psychiatric symptoms, could be reduced by physical activity interventions [31, 45]. Psychiatric disorders are often associated with elevated levels of stress and inflammatory biomarkers while physical activity has an anti-inflammatory effect. Therefore, stress and inflammatory biomarkers are potential mechanisms through which physical activity reduces psychiatric symptoms [16]. Similarly, adrenal hormones and neurotransmitters, such as serotonin, are other potential neurobiological mechanisms that may be stimulated by physical activity, and in turn, reduce depressive symptoms [38, 43]. Last, physical activity may also affect psychiatric symptoms through epigenetic mechanisms [55]. For instance, Liao et al. [47] explored the possibility that BDNF methylation could mediate the effect of physical activity on depressive symptoms in older people and found preliminary evidence for this hypothesis. Overall, although there are promising and highly accepted neurobiological mechanisms in the field, more studies are needed before we can establish the role of specific neurobiological mechanisms in the relationship between physical activity and psychiatric symptoms across the lifespan.

### Limitations and Strengths

This review only included 22 studies that fit our inclusion criteria, and hence, might not include all potential mechanisms linking physical activity with psychiatric symptoms. Because of the heterogeneity of studies, we also could not perform a meta-analysis to thoroughly synthesize the current body of evidence.

Furthermore, we strived to include articles with all types of physical activity in this review, and hence, did not make any distinctions between physical activity, exercise, and sport participation. Vella et al. [51] proposed five main qualitative characteristics of physical activity: type of activity, delivery, social environment, physical activity, and domain. The effects of different activities may be different owing to the qualitative characteristics of each activity. For example, sport participation usually includes the social component that may not present in some types of physical activity and should be taken into account in future studies.

On the other hand, this review has a few strengths. As there were many reviews focused on specific populations, we aimed at a summary of the existing literature by including healthy participants of all ages and all types of studies to provide an overview of the current body of research. Additionally, we strived for open practice in scientific research by registering this review a priori in the PROSPERO database and publishing the protocol of this review [21]. Finally, we also followed the PRISMA guidelines for a systematic review and included articles across five electronic databases (Table S1 of the ESM).

### Literature Gaps and Future Research

- While we aimed to investigate the mechanisms through which physical activity may influence externalizing and internalizing symptoms, we failed to identify any studies examining the effect of physical activity on externalizing symptoms.
- The variety of measurements available for assessing depressive and anxiety symptoms may pose as an obstacle for research and clinical application. Researchers should consider using similar measures when assessing symptoms of the same disorder.
- Only three mechanisms (i.e., self-esteem, self-concept, self-efficacy) were sufficiently studied. Therefore, further research is needed to explore other paths.
- As most studies focused on children and adolescents, there is a need for additional studies examining potential mechanisms in adult and elderly populations.
- Integrated studies that examine the combined and independent contributions of neurobiological and psychosocial mechanisms are needed to obtain the overall picture.

## Conclusions

The findings from our systematic review suggest that self-esteem, self-concept, and self-efficacy are potential paths through which physical activity reduces psychiatric symptoms (specifically depressive and anxiety symptoms) across the lifespan. Therefore, future interventional studies should consider incorporating these mechanisms to develop more effective interventions. There were insufficient studies to establish the role of other psychosocial and neurobiological mechanisms linking physical activity with psychiatric symptoms across the lifespan.

### Supplementary Information

Below is the link to the electronic supplementary material.

Supplementary file1 (DOCX 41 kb)
